# Pulmonary Complications After Pediatric Stem Cell Transplant

**DOI:** 10.3389/fonc.2021.755878

**Published:** 2021-10-13

**Authors:** Taylor Fitch, Kasiani C. Myers, Maya Dewan, Christopher Towe, Christopher Dandoy

**Affiliations:** ^1^ Division of Bone Marrow Transplantation and Immune Deficiency, Cincinnati Children's Hospital Medical Center (CCHMC), University of Cincinnati School of Medicine, Cincinnati, OH, United States; ^2^ Division of Critical Care, Cincinnati Children's Hospital Medical Center (CCHMC), University of Cincinnati School of Medicine, Cincinnati, OH, United States; ^3^ Division of Pulmonology, Cincinnati Children's Hospital Medical Center (CCHMC), University of Cincinnati School of Medicine, Cincinnati, OH, United States

**Keywords:** pulmonary complications, hematopoietic stem cell transplant, pediatric, non-infectious complications, infectious complications

## Abstract

The number of disorders that benefit from hematopoietic stem cell transplantation (HSCT) has increased, causing the overall number of HSCT to increase accordingly. Disorders treated by HSCT include malignancy, benign hematologic disorders, bone marrow failure syndromes, and certain genetic diagnoses. Thus, understanding the complications, diagnostic workup of complications, and subsequent treatments has become increasingly important. One such category of complications includes the pulmonary system. While the overall incidence of pulmonary complications has decreased, the morbidity and mortality of these complications remain high. Therefore, having a clear differential diagnosis and diagnostic workup is imperative. Pulmonary complications can be subdivided by time of onset and whether the complication is infectious or non-infectious. While most infectious complications have clear diagnostic criteria and treatment courses, the non-infectious complications are more varied and not always well understood. This review article discusses pulmonary complications of HSCT recipients and outlines current knowledge, gaps in knowledge, and current treatment of each complication. This article includes some adult studies, as there is a significant paucity of pediatric data.

## Introduction

Each year, approximately 2,500 children in the United States undergo hematopoietic stem cell transplantation (HSCT) for malignant and non-malignant conditions (1, 2). Over the past few years, improvements in supportive care have improved outcomes; however, pulmonary complications continue to be a major cause of morbidity and mortality in children undergoing autologous and allogeneic HSCT (3, 4). This article will review the causes of pulmonary complications post-HSCT, evaluate the workup and diagnosis of patients with respiratory symptoms, discuss the management of pulmonary complications, and outline the future direction of our understanding of these complications.

### Incidence of Pulmonary Complications

Pulmonary complications occur in approximately 25% and 27% of pediatric and adult HSCT recipients, respectively, and are a leading cause of transplant-related mortality (5). Overall, about 9% of HSCT recipients require invasive ventilation and 10% require non-invasive ventilation (4–11). However, it is likely that pulmonary complications are underreported in both adult and pediatric patients. Roychowdhury et al. (12) reviewed autopsies and bronchoalveolar lavage (BAL) slides of patients who died after HSCT and determined that pulmonary complications occurred in 40 (80%) of the 50 cases and were a major contributor of transplant-related mortality in 74% (37 of 50). Furthermore, Inaba et al. (13) evaluated the incidence of abnormal pulmonary function tests in 89 transplant survivors and demonstrated that abnormal pulmonary function testing was seen in 40.4% of baseline testing and 64% of post-HSCT testing.

### Timing of Pulmonary Complications After Hematopoietic Stem Cell Transplantation

Time of onset from cell infusion is helpful when evaluating causes of pulmonary complications: pre-engraftment (0–30 days), post-engraftment (30–100 days), and late phase (over 100 days) (14). The pre-engraftment period is marked by neutropenia, mucositis, indwelling lines, and acute graft-versus-host disease (GVHD) (14–17). Post-engraftment phase is marked by impaired cellular immunity and acute GVHD. The late phase is marked by impaired humoral/cellular immunity and chronic GVHD (14, 17, 18). In all phases, severe GVHD (II–III), acute or chronic, increases relative risk of pulmonary complications. One study notes an increase in relative risk of 2 with a 95% CI of 1.1–3.7 (4, 19).

### Further Classification

Pulmonary complications after HSCT can be subclassified as infectious vs. non-infectious (**Table 1**), related to the way impairment of immune function differs over time after HSCT. Pulmonary complications caused by infectious vs. non-infectious etiologies have incidence ranging from 13.9%–54.8% to 10.2%–39.7%, respectively.

**Table 1:** Infectious and non-infectious etiologies of pulmonary complications after stem cell transplant further subdivided by time after transplant.

	Pre-Engraftment (Days 0–30)	Post-Engraftment (Days +30–100)	Late phase (over 100 days)	
**Infectious Diagnoses**	**Bacterial** Gram-negative organisms Gram-positive organisms **Fungal** Candida **Viral** Community-acquired viral pneumonia (e.g., influenza) *Cytomegalovirus*	**Bacterial** Gram-positive organisms Gram-negative organisms **Fungal** Candida Aspergillosis Mucormycosis PJP **Viral** Community-acquired viral pneumonia (e.g., influenza) *Cytomegalovirus*	**Bacterial** Gram-positive organisms Gram-negative organisms Encapsulated bacteria *Nocardia* *Mycobacterium* **Fungal** Aspergillosis Mucormycosis PJP **Viral** Community-acquired viral pneumonia (e.g., influenza) *Cytomegalovirus*
Non-infectious Diagnoses	Pulmonary hypertension Drug toxicity Peri-engraftment syndrome Diffuse alveolar hemorrhage Idiopathic pneumonia syndrome Pulmonary alveolar proteinosis	Drug toxicity Diffuse alveolar hemorrhage Cryptogenic-organizing pneumonia Idiopathic pneumonia syndrome Pulmonary hypertension	Cryptogenic-organizing pneumonia Bronchiolitis obliterans Pulmonary hypertension Drug toxicity

PJP, Pneumocystis jirovecii pneumonia.

## Infectious Pulmonary Complications

Infectious pulmonary complications occur at higher rates in both allogeneic and autologous HSCT (**Table 2**) (5, 15, 17, 21, 22). Infectious complications are higher in pre-engraftment and post-engraftment phases and occur in 20%–30% of patients. This increased risk is related to the impairment of immunological function due to proximity of conditioning regimen and stem cell infusion (14, 15, 21, 23). One study evaluating autologous HSCT demonstrated infectious etiology of pulmonary complications at an incidence of 13.9%, non-infectious at 10.2%, and patients experiencing both at 3.5% (5). Bacterial, viral, and fungal infections all contribute to pulmonary complications after HSCT.

**Table 2:** Infectious etiology summary (20).

Diagnosis	Laboratory Testing	Imaging	Treatment
**Bacterial**			
Gram-negative organisms	-BAL fluid culture -Blood cultures with imaging findings	CXR/CT chest: consolidation or pleural effusion	-Antibiotics based on biogram or sensitivities -Cephalosporins -Aminoglycosides - Fluoroquinolones -Carbapenems
Gram-positive organisms	-BAL fluid culture -Blood cultures with imaging findings	CXR/CT chest: consolidation or pleural effusion	-Antibiotics based on biogram or sensitivities -Cephalosporins -Vancomycin -Daptomycin -Penicillins
Encapsulated organisms	-BAL fluid culture -Blood cultures with imaging findings	CXR/CT chest: consolidation or pleural effusion	-Antibiotics based on biogram or sensitivities -Cephalosporins -Penicillins -Fluoroquinolones
*Nocardia*	-BAL fluid culture -Blood culture	CT chest: lobular and multinodular infiltrates, reticulonodular infiltrates	Sulfonamide -Trimethoprim-sulfamethoxazole
**Fungal**			
Aspergillosis	-BAL galactomannan -Serum galactomannan and aspergillosis serum PCR	CT chest: perinodular halos with ground-glass opacities	Antifungal based on sensitivities Azoles -Voriconazole -Posaconazole Polyenes -Amphotericin B
Candidiasis	-BAL fungal cultures -Serum fungal cultures	CT chest: tree-in-bud changes, ground-glass opacities, cavitation	Antifungal based on sensitivities Echinocandins -Micafungin Azoles -Voriconazole -Posaconazole
Mucormycosis	-BAL fungal cultures -Serum fungal cultures	CT chest: area of central ground glass necrosis surrounded by a ring of consolidation	Polyenes -Amphotericin B Surgical resection
**Viral**			
Cytomegalovirus	BAL: CMV PCR Serum: CMV PCR	CT chest: ground-glass opacities, air-space consolidations, and reticulonodular patterns	Purine nucleosides -Ganciclovir -Valganciclovir Pyrophosphate analog -Foscarnet -Cidofovir
Adenovirus	-BAL: Adenovirus PCR -Serum: Adenovirus PCR	CT chest: bilateral ground-glass opacities with a random distribution	Pyrophosphate analog -Cidofovir Immunoglobulins
Community-acquired viral pneumonia (RSV, HMPV, rhinovirus, and parainfluenza)	-BAL: Respiratory viral PCRs -Nasal swab: Respiratory viral PCRs	CT chest: multifocal patchy consolidation with ground-glass opacities. Can have centrilobular nodules with bronchial wall thickening	Supportive care Immunoglobulins
COVID-19	-BAL: COVID PCR testing -Nasal swab: Respiratory viral PCRs -Serum: Antibody testing	CT chest: Bilateral ground-glass opacities with peripheral distribution, consolidations, linear opacities, septal thickening, halo sign	Supportive care Steroids ± Remdesivir ± Tocilizumab

Infectious etiologies of pulmonary complications. Describes diagnostic testing, imaging results, and most commonly used treatments, though there is institutional variation. BAL, bronchoalveolar lavage; CMV, Cytomegalovirus; RSV, respiratory syncytial virus; HMPV, human metapneumovirus.

### Bacterial Infections

Bacterial pneumonia is the most prevalent type of infectious complication in all phases, with an incidence up to 45% (15, 21, 23, 24). In the pre-engraftment phase, the major causative agents include Gram-negative organisms (such as *Pseudomonas aeruginosa*) due to the poor mucosal barrier, acute GVHD, central lines, and protracted neutropenia (14, 15, 22, 25). In the late phase, infectious etiologies more commonly feature encapsulated organisms and are associated with chronic GVHD. This is likely due to continuation of immunosuppressive medications (14, 15, 25, 26).

*Nocardia* infection has a low cumulative annual incidence. One study noted a cumulative annual incidence of 1.75% throughout the course of treatment, predominantly occurring in the late phase (15, 27–29). Nocardial infection is more common in patients with allogeneic HSCT, those with a history of acute GVHD, and those actively being treated for chronic GVHD at the time of diagnosis (27, 28). Additional risk factors include other concurrent infections, in particular, CMV infection (27, 28). *Nocardia* tends to be disseminated at diagnosis but commonly has a pulmonary locus (30). There is some evidence that those who receive pentamidine prophylaxis for *Pneumocystis jirovecii* also have increased risk, but this is not consistently demonstrated throughout the literature (28, 30).

*Mycobacterium* infections including both tuberculosis and non-tuberculous subtypes occur at low incidence worldwide (0.1%–5.5%) and are more prevalent in those who have received an allogeneic HSCT. In the United States, incidence has been reported from 0.0014% to 3% (31–33). Infection typically occurs in the late phase, and while *Mycobacterium* infections can be disseminated, infection is predominantly in the lungs (31, 32). Tuberculosis infection is associated with older age and chronic GVHD (34). Most recommendations are for conservative management, with treatment if the patient has a tuberculosis exposure even with negative skin testing. Currently, there is no evidence for prophylaxis as incidence remains low (32). Studies have demonstrated that it is likely safe to treat after Day +100, and treatment consists of isoniazid, rifampin, ethambutol, and pyrazinamide for 6–9 months (33).

### Fungal Infections

Overall, the reported incidence of fungal infections ranges from 4% to 34%, occurring most commonly in allogeneic HSCT patients and during the post-engraftment and late phases. Mortality can be up to 33.3% (3, 4, 17, 23). The most common fungal pulmonary complications in HSCT patients are invasive aspergillosis, followed by invasive candidiasis, then mucormycoses (17, 35). In one large multicenter study, incidence rates of each fungal infection were reported as 43%, 28%, and 8% respectively (35). There is an increase in fungal infections with protracted/continued neutropenia (60 days or longer) and concurrent GVHD (35).

Invasive aspergillosis has a reported incidence in autologous HSCT of 1% to 5%, most frequently diagnosed in post-engraftment and late phases (15, 35). Invasive aspergillosis cases have continued to decline with the integration of granulocyte colony-stimulating factor and azole prophylaxis in treatment (14, 25, 36). Aspergillosis is diagnosed using a combination of radiologic and serologic testing. Serum galactomannan and aspergillosis serum PCR testing can be sent for diagnosis, but the most sensitive/specific test is the BAL galactomannan (14, 25, 37). The accuracy of testing has been shown to be related to neutrophil count and underlying condition (38). The current recommended treatment is with antifungals such as voriconazole or amphotericin B (25, 37).

Overall, invasive candidiasis infections have been decreasing in incidence, in particular, *Candida albicans*. There has been an increase in the rate of *Candida glabrata* and *Candida krusei*, likely secondary to antifungal prophylaxis (15, 39); one study demonstrated that 70% of infections were attributed to that non-albican species when analyzing autologous HSCT recipients with *Candida* infections (35). *Candida* infections span the entire course of transplant, peaking in the post-engraftment phase before the first 120 days (35). Diagnosis is made through fungal cultures from both serum and BAL. Initial treatment of choice for *Candida* species is echinocandins (such as micafungin) or voriconazole, with further modification based on sensitivities of cultures (40).

Mucormycosis infections are also increasing in incidence with the use of azole antifungal prophylaxis (25, 41). One study showed an incidence of 8% with infections typically occurring in the late phase (35). CT scans can be helpful for diagnosis and can show reversed halo signs (an area of central ground-glass necrosis surrounded by a ring of consolidation); however, for diagnosis, BAL with fungal cultures are needed. Treatment is with amphotericin B and accompanied by surgical resection if without significant morbidity/mortality (14, 25, 41).

Of note, *P. jirovecii* pneumonia is a rare complication after HSCT, with an incidence of 0.63% in allogeneic HSCT and 0.28% in autologous HSCT recipients (42). In one study, when a patient was diagnosed, they were 6.87 times more likely to die when compared to their matched controls (42). Patients are at an increased risk if they have GVHD and/or poor immune reconstitution (42). As this complication is a rare cause of pulmonary complications, it will not be discussed in significant detail in this review.

### Viral Infections

Viral pneumonia in all HSCT patients ranges between 4% and 21.9%, with a greater incidence in allogeneic HSCT patients. The most commonly reported viral infections are *Cytomegalovirus* (CMV) and adenovirus (17, 21, 23). In a study evaluating the changes in rates of infection over time, CMV disease fell from 8% to 5% from 1993–1997 compared to 2003–2007 (3). Mortality greatly varies between types of viral infection, viral quantities, and autologous vs. allogeneic HSCT. Respiratory syncytial virus (RSV), influenza, and parainfluenza are other common etiologies of viral pneumonia that are seen in HSCT patients (14).

CMV becomes a major cause of pneumonia starting at 3 weeks posttransplant and continues into the late phase. There have been improvements in infection rates through careful selection of donors, careful serology monitoring, and early intervention. The greatest predictor of CMV infection is the serology status of the recipient (14, 15, 23, 25, 43, 44). Autologous HSCT patients have a reported incidence of 1%–9% (15, 44). Again, acute GVHD and allogeneic HSCT patients have an increased risk of CMV infection (14, 43). Diagnosis requires radiologic and positive CMV PCR from BAL or viral cultures (14, 15). Mortality still approached 31% in one study focusing specifically on autologous HSCT recipients. While letermovir can be used for prophylaxis, it has not been used to treat active infections (45, 46). Treatment of CMV infection includes gancyclovir and foscarnet with/without CMV immunoglobulins (14, 15, 44). Resistance testing can be performed if continued breakthrough viremia or increasing viral count while on treatment. Some centers treat more persistent or severe infections with virus-specific T cells (targeted therapy) (14).

While a common community-acquired entity, RSV infection in HSCT patients (as well as rhinovirus, parainfluenza, and human metapneumovirus) has been shown in studies to progress to pneumonia between 35% and 58% of the time (15, 47, 48). These infections occur equally in autologous and allogeneic HSCT (15). Additional risk factors for developing a significant pneumonia from these common viruses include severe lymphopenia, T cell-depleting or myeloablative conditioning, and acute GVHD (47, 48). RSV can be particularly severe in HSCT patients, leading to additional complications such as acute respiratory distress syndrome or even diffuse alveolar hemorrhage (DAH) that results in invasive respiratory support (4, 49, 50). One study reported that up to 10% of their patients developed acute lung injury from RSV (50). Chemoprophylaxis with palivizumab in high-risk children (RSV outbreak or young infants) has been shown to have some benefit in prevention of RSV infection (47). Rapid RSV PCR is diagnostic, and in severe cases, aerosolized ribavirin and immunoglobulins can be considered, though there are little data to support an improvement in mortality (14, 25, 49).

Other viral infections that should not be excluded from evaluation are human herpesvirus 6 (HHV6), herpes simplex virus (HSV), and adenovirus. Going forward, additional studies will be needed on the novel coronavirus disease 2019 (COVID-19) virus in HSCT patients.

## Non-Infectious Complications

While there has been significant reduction in developing infectious complications secondary to improved prophylaxis, improved diagnostic testing, and targeted antimicrobials, there has been no significant improvement in the incidence of non-infectious pulmonary complications in HSCT (**Table 3**) (8, 15, 51, 52). Two studies evaluating allogeneic and autologous HSCT show non-infectious pulmonary complications with an incidence of 28% and 10.2%, respectively (5, 17). Some main categories of non-infectious pulmonary complications in HSCT include peri-engraftment respiratory distress syndrome (PERDS), idiopathic pneumonia syndrome (IPS), DAH, drug toxicity, cryptogenic organizing pneumonia (COP), bronchiolitis obliterans (BO), and pulmonary veno-occlusive disease. These complications also have a typical time of presentation after stem cell infusion, which will be further discussed below. Diagnosis is often difficult due to significant overlap, poor diagnostic confirmatory testing, and increased risk of invasive procedure to identify the underlying etiology (8, 51, 52). This highlights the need for studies to further investigate and better determine the mechanism of these injuries, possible preventative measures, and elucidation of better treatment options for these complications.

**Table 3:** Non-infectious causes of pulmonary dysfunction.

Diagnosis:	Laboratory Testing	Imaging	Treatment
Engraftment syndrome/PERDS	- No definitive laboratory testing	- CXR/CT chest - Pulmonary edema	- Corticosteroids - Diuretics - Supportive care
IPS	-BAL fluid without signs of infectious process	-CXR/CT chest: multilobular infiltrates	IV corticosteroids -TNF alpha-binding protein -Supportive care
DAH	-BAL fluid: –hemosiderin-laden macrophages –Increasingly bloody samples	-CT chest: Lobular/lobar ground-glass opacities. Prominent segmental bronchi	-IV corticosteroids -Supportive care
Drug toxicity	-BAL fluid: without signs of infectious process -**Lung biopsy: hypersensitivity reaction with eosinophilic pneumonia	-CT chest: patchy ground-glass opacities, sometimes with septal thickening	-IV corticosteroids -Supportive care
COP	-Lung biopsy: patchy plugs of granulation tissues filling lumens of distal airways. Chronic interstitial inflammation and no prominent bronchiolar damage	-CT chest: patchy consolidations with elongated distribution and ground-glass opacities	-Corticosteroids (systemic)
PHTN	-None	-ECHO: increased pulmonary vascular resistance and elevated right ventricular pressure	-Supportive care -Oxygen therapy -Inhaled nitric oxide -Calcium channel blockers -Phosphodiesterase-5 inhibitors
BO	-Lung biopsy: constrictive bronchiolitis and submucosal bronchiolar fibrosis	-CT chest: small airway thickening or bronchiectasis -PFTs: Obstructive pattern, not responsive to albuterol	-Corticosteroids (inhaled and systemic) -TNF-alpha modulators

Describes diagnostic testing, imaging results, and most commonly used treatments, though there is institutional variation. BAL, bronchoalveolar lavage; PERDS, peri-engraftment respiratory distress syndrome; IPS, idiopathic pneumonia syndrome; DAH, diffuse alveolar hemorrhage; COP, cryptogenic organizing pneumonia; PHTN, pulmonary hypertension; BO, bronchiolitis obliterans; PVOD, pulmonary veno-occlusive disease; IV, intravenous; TNF, tumor necrosis factor.

**Not routinely performed.

### Peri-Engraftment Respiratory Distress Syndrome

PERD typically occurs within the first 5 days of engraftment (pre-engraftment phase) and is accompanied by established clinical criteria, with pulmonary edema seen on imaging (25). Clinical criteria include fever, rash, hepatic or renal dysfunction, weight gain, hypoxemia, and transient encephalopathy (15, 53). The etiology is not completely understood but thought to be secondary to pro-inflammatory cytokines (54). PERD does have a higher incidence in autologous HSCT patients, with studies showing an incidence between 2.5% and 20% (5, 14, 54, 55). Overall incidence seemed to increase over time, which is thought to be secondary to the introduction of granulocyte colony-stimulating factor during HSCT (56). Typically, there is a good response to steroids (14, 15, 53).

### Idiopathic Pneumonia Syndrome

IPS usually presents with fever, acute respiratory distress, and alveolar damage that has an unknown underlying etiology (not caused by infection or end organ damage) (8, 51, 52). Currently, the best evaluation of etiology has come from murine models that suggest conditioning regimens including lung irradiation, cyclophosphamide, busulfan, or previous treatments with carmustine (BCNU), etoposide, bleomycin, and cisplatin all increased the risk of epithelial injury. This leads to activation of pulmonary macrophages and alloreactive T lymphocytes. Implicated cytokines include interleukin-6, interleukin-8, and in particular tumor necrosis factor (TNF)-alpha (52, 56, 57). Imaging is non-specific with multilobular infiltrates. BAL is typically performed to rule out an underlying infectious process (52, 58). Onset usually is in the later portion of pre-engraftment to the beginning of the post-engraftment phase (21, 51). There is a higher incidence in allogeneic HSCT with a mean up to 10% in allogeneic HSCT patients and 5.8% in autologous HSCT patients (14, 25, 51, 58). The onset for autologous HSCT is typically later in the mid-to-late post-engraftment phases (21, 58). Risk factors specifically for developing IPS include chest irradiation, older age, being female, or solid tumor diagnosis (58). Other risk factors that seem to increase the incidence of IPS include high-dose cyclophosphamide and adding busulfan to the conditioning regimen (52). Mortality is significant from 60%–80% in allogeneic transplants, with few recent studies specifically looking at mortality in autologous transplant patients (58). The patients who develop this complication have the highest rate of mortality once intubated (approaching 74%) (59).

Currently, first-line treatment with corticosteroids and supportive care is recommended; however, studies are mixed whether there is improvement in respiratory support or outcome (52, 59). Etanercept (TNF alpha binding protein), has been shown to reduce pulmonary vascular endothelial cell apoptosis; one study evaluating the combination of steroids and Etanercept demonstrated improvement in mortality, increasing D +28 survival to 73% (15, 57, 60). Thus, a randomized, double-blind, placebo-controlled trial was initiated comparing etanercept to placebo; all patients received methylprednisolone. This study was unable to produce significant results in mortality/outcome; however, the trial’s results are difficult to interpret as it was halted due to poor study enrollment (25, 61). This demonstrates again an area that necessitates further investigation to find alternative treatment options and evaluate ways to improve outcomes. Treatment with agents such as etanercept should only occur after confirmation that there is not an underlying infectious etiology. IPS additionally encompasses subcategories including DAH and COP, which will be discussed later.

### Diffuse Alveolar Hemorrhage

DAH is a subcategory of IPS that is defined by hemorrhagic alveolitis. In pediatric and adult allogeneic HSCT, incidence of DAH ranges from 5% to 12% with a median onset of 19 days as compared to autologous HSCT recipients where DAH incidence ranges from 2.1% to 12%, with a median time of onset of 12 days (5, 58, 62, 63). Definitive diagnosis requires a BAL sample with at least 20% of hemosiderin-laden macrophages, blood in 30% of alveolar surfaces, with increasingly bloody samples (15, 58, 64). In one study, DAH was associated with engraftment, an age over 40, solid malignancies, high fevers, severe mucositis, and/or with renal insufficiency (65). Currently, treatment recommendations include high-dose steroids and supportive care (5, 62). Small studies have been performed in patients with DAH (both transplant induced and not) that have evaluated inhaled/nebulized tranexamic acid ± recombinant activated factor VII with reported success; however, further investigation with a larger sample size is required before integrating this into standard of care (63, 66, 67). Despite early intervention and supportive care, mortality historically has been up to 80%–100% in allogeneic HSCT patients. Newer studies suggest that mortality for autologous HSCT patients is closer to 28% and 70% for allogeneic patients (21, 58, 62, 64, 67). If DAH occurs in the first 30 days, mortality is significantly lower (64).

### Drug Toxicity/Delayed Pulmonary Toxicity Syndrome

Drug toxicity has a varying degree of severity—from mild dyspnea to respiratory failure requiring mechanical ventilation. Incidence ranges from 22% to 49% in autologous HSCT patients with a mean onset at Day +45 (post-engraftment phase); however, there is a wide range from 21 to 149 days (52, 58 68). Drug toxicity is seen more frequently in patients who have had regimens including BCNU, etoposide, cyclophosphamide, bleomycin, and cisplatin (52, 58, 68, 69). Radiologically, CT scans are not specific but can demonstrate patchy ground-glass opacities, sometimes with septal thickening. Biopsy can show hypersensitivity reaction with eosinophilic pneumonia or, if performed later, a thickening of the interstitium with early fibrosis (51, 52). The treatment of choice is corticosteroids, though little data are available on the overall mortality of this complication (58).

### Cryptogenic Organizing Pneumonia

COP tends to be a more subacute process with fever, dyspnea, and cough (51). Incidence typically ranges from 0.9% to 10% and can occur in both allogeneic and autologous HSCT patients (14, 15, 51). For allogeneic patients, there is a well-described association with chronic GVHD, thus suggesting an immune-mediated response (51). For autologous HSCT patients, the proposed mechanism is secondary to underlying infection or drug toxicity (51). COP usually occurs within the first 100 days and occurs more frequently in allogeneic HSCT patients. On chest CT, patchy consolidations with elongated distribution and ground-glass opacities can be seen (14, 51, 70). Diagnosis is confirmed *via* biopsy that shows patchy plugs of granulation tissues that fill lumens of distal airways (alveoli) with chronic interstitial inflammation and no prominent bronchiolar damage (15, 52). Corticosteroids remain the treatment of choice with a slow taper, usually with significant improvement. In one study, 78%–80% of patients demonstrated a good response (14, 15, 52, 71). However, there are no studies looking at what duration of steroid therapy is most appropriate. Additionally, COP tends to have a widely varied reported relapse rate of 9%–58%, which is likely secondary to no standardization of treatment across institutions (52, 70).

### Pulmonary Hypertension

Pulmonary hypertension (PHTN) is characterized by increased pulmonary vascular resistance and elevated right ventricular pressure (72). The incidence of PHTN is not well defined but has been estimated between 15% and 28% with a mortality up to 55%–86% (73, 74). This complication most commonly occurs in the late phase with a reported median of Day +70; however, the range is wide from Day 0 to +365 (72). The underlying etiology in HSCT is not well understood; the proposed mechanism is endothelial injury in both pre/post-pulmonary capillary vasculature, leading to smooth muscle proliferation, fibroblast infiltration, and ultimately hypertrophy of the vasculature (72, 75). This damage can be instigated by other underlying disorders such as thrombotic microangiopathy and atypical hemolytic uremic syndrome (72, 73). Chemotherapy agents that have been implicated include mitomycin, bleomycin, cisplatin, vincristine, cyclophosphamide, and BCNU (76). Additional risk factors include high-dose preparative chemotherapy and radiation prior to transplantation. Diagnosis is usually obtained *via* electrocardiogram and echocardiogram, but sometimes more invasive measures are implemented, such as transesophageal echocardiology or cardiac catheterization (72, 75). Biopsy can be obtained to confirm the diagnosis and shows widespread fibrous proliferation in the pulmonary venules and small veins; however, this is not typically performed due to the morbidity and mortality associated with the procedure (76). Treatment includes supportive care, oxygen therapy, inhaled nitric oxide, calcium channel blockers, and phosphodiesterase-5 inhibitors (72). For long-term damage caused by PHTN, high-dose steroids can be used, though there are no studies evaluating the effectiveness of this treatment (76, 77). There are limited studies evaluating this complication, and further investigation is warranted.

### Bronchiolitis Obliterans

BO exclusively occurs in allogeneic HSCT patients and is one of the most common causes of late-phase complications (from Day +90 to 2 years) (15, 25, 51). Incidence ranges from 2% to 48%; this wide range is likely secondary to the inconsistencies in diagnostic criteria between studies (51). Associated features include acute GVHD, older age, non-related donor, total body irradiation, peripheral stem cell source, and busulfan-based conditioning regimen (14, 25, 51, 52). BO is a histologic diagnosis that can be made after lung biopsy, which shows constrictive bronchiolitis and submucosal bronchiolar fibrosis (15, 25, 51, 78). However, lung biopsies are not without significant morbidity and mortality. Thus, clinical criteria have been established through the National Institutes of Health Consensus Development Project to describe bronchiolitis obliterans syndrome (BOS). This requires four features to meet diagnostic criteria. First, FEV1/VC <0.7 (or 5th percentile of predicted). Second, FEV1 <75% of predicted with ≥10% decline over less than 2 years. FEV1 should not correct >75% with albuterol. Third, no identified infection. Fourth, evidence of air trapping/bronchiectasis on high-resolution chest CT or residual volume >120% of predicted value (79). While this is a useful outline, studies have shown that not all patients with histologic BO meet the established criteria of BOS. Treatment can include both inhaled and systemic steroids with other immunosuppressive medications, though very few studies demonstrated significant clinical benefit (51). For patients whose symptoms are steroid refractory, treatment can include Janus-associated kinase 1/2 inhibitors such as ruxolitinib (80, 81). Studies have been published reporting 59%–68% overall response rate with a tolerable safety profile (80, 82). Most common toxicities included reactivation thrombocytopenia and anemia; some studies have reported increased CMV reactivation, though this is not consistently demonstrated across all studies (80–83). However, these studies evaluated those 12 years of age or older; studies evaluating effectiveness/dosing in children younger than 12 and dosing are ongoing (81, 83). Other agents that are currently under investigation include belumosudil and ibrutinib, though further investigation is needed for use in pediatrics (84, 85). Despite aggressive treatment, most patients have acute flares with mortality rate being between 12% and 27% at 5 years due to secondary infection or respiratory complications (8, 51, 78).

Other entities that can cause pulmonary complications that are not discussed here include acute GVHD, pulmonary alveolar proteinosis, pulmonary cytolytic thrombi, and chronic GVHD.

## Timeline/Classification of Pulmonary Complications Posttransplant

HSCT is associated with a variety of pulmonary complications that can be classified by time after stem cell infusion (**Tables 4**, **5**). Risk for developing pulmonary complications varies greatly by type of transplant (allogeneic vs. autologous), previous treatments (chemotherapy, radiation), and underlying demographics of the patient (age and primary diagnosis). Despite our increased knowledge surrounding pulmonary complications, arriving at the correct diagnosis requires thorough and often invasive evaluation.

**Table 4:** Infectious complications and the most common times in which complication develops.

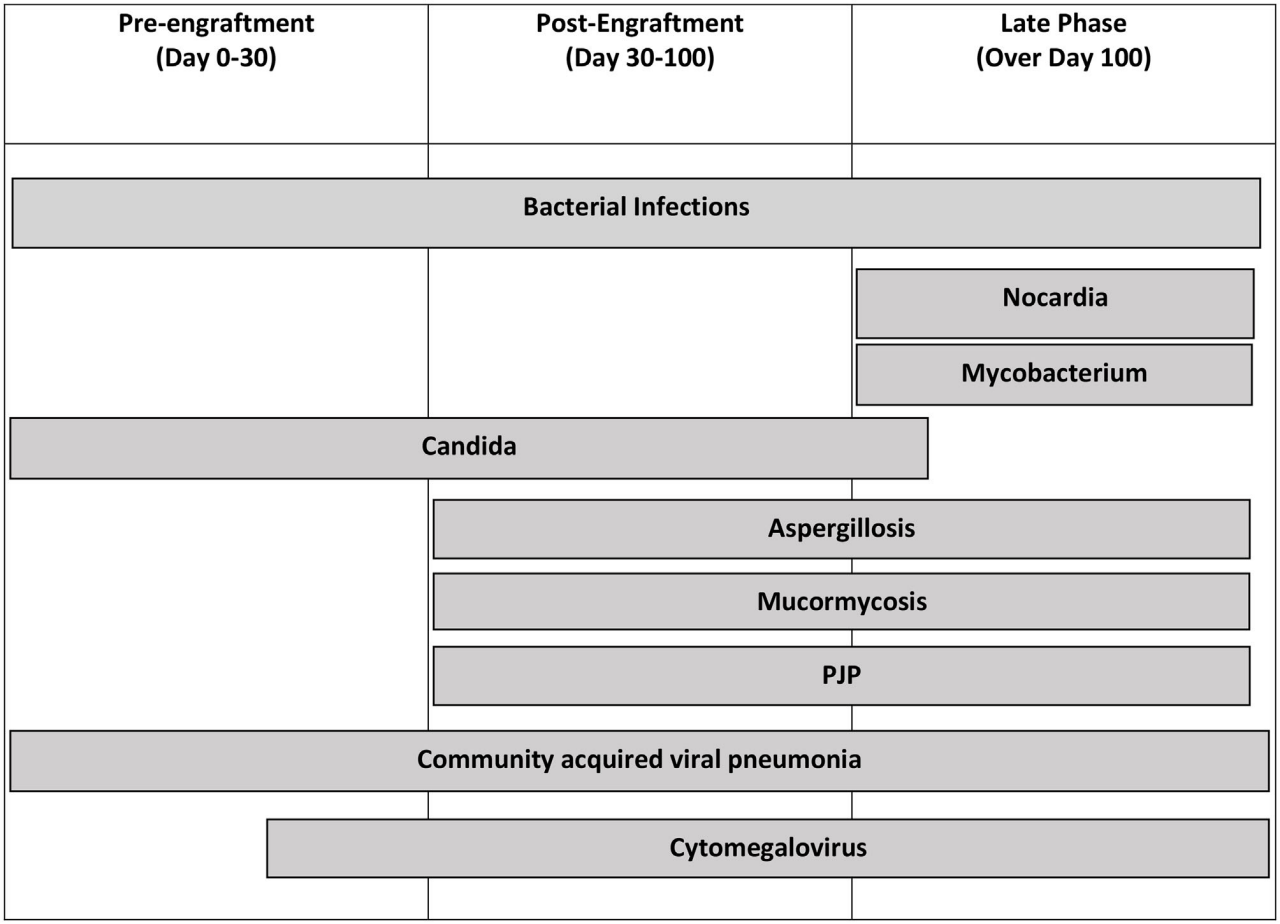

**Table 5:** Non-infectious complications and the most common times in which complication develops.

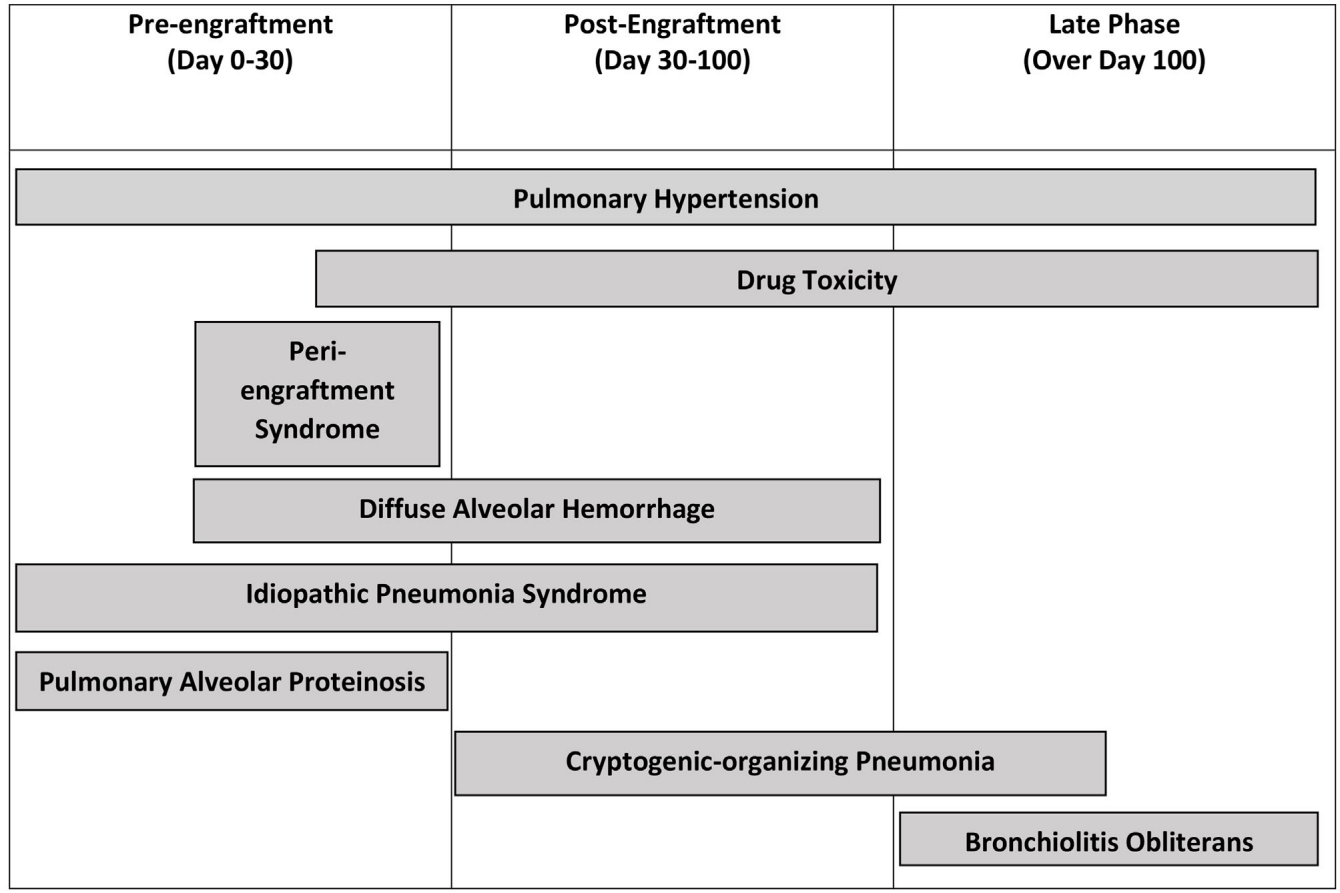

## Diagnosis of Pulmonary Complications

In the acute phase, initial evaluation starts with imaging (**Table 6**). Chest X-rays are typically performed and can be helpful if an infiltrative process is found, which would indicate a BAL is needed for better evaluation (5, 23). However, chest X-rays can be normal in 15% of patients, and thus, if clear, it is prudent to proceed with a chest CT scan (5). Chest CT scans while sometimes suggestive are rarely diagnostic (25). Ultimately, patients most often require bronchoscopy with BAL or even lung biopsy to finalize diagnosis. Of note, these interventions have better diagnostic yield after imaging has been obtained (5).

**Table 6:** Diagnostic Algorithm of Pulmonary Complications after HSCT.

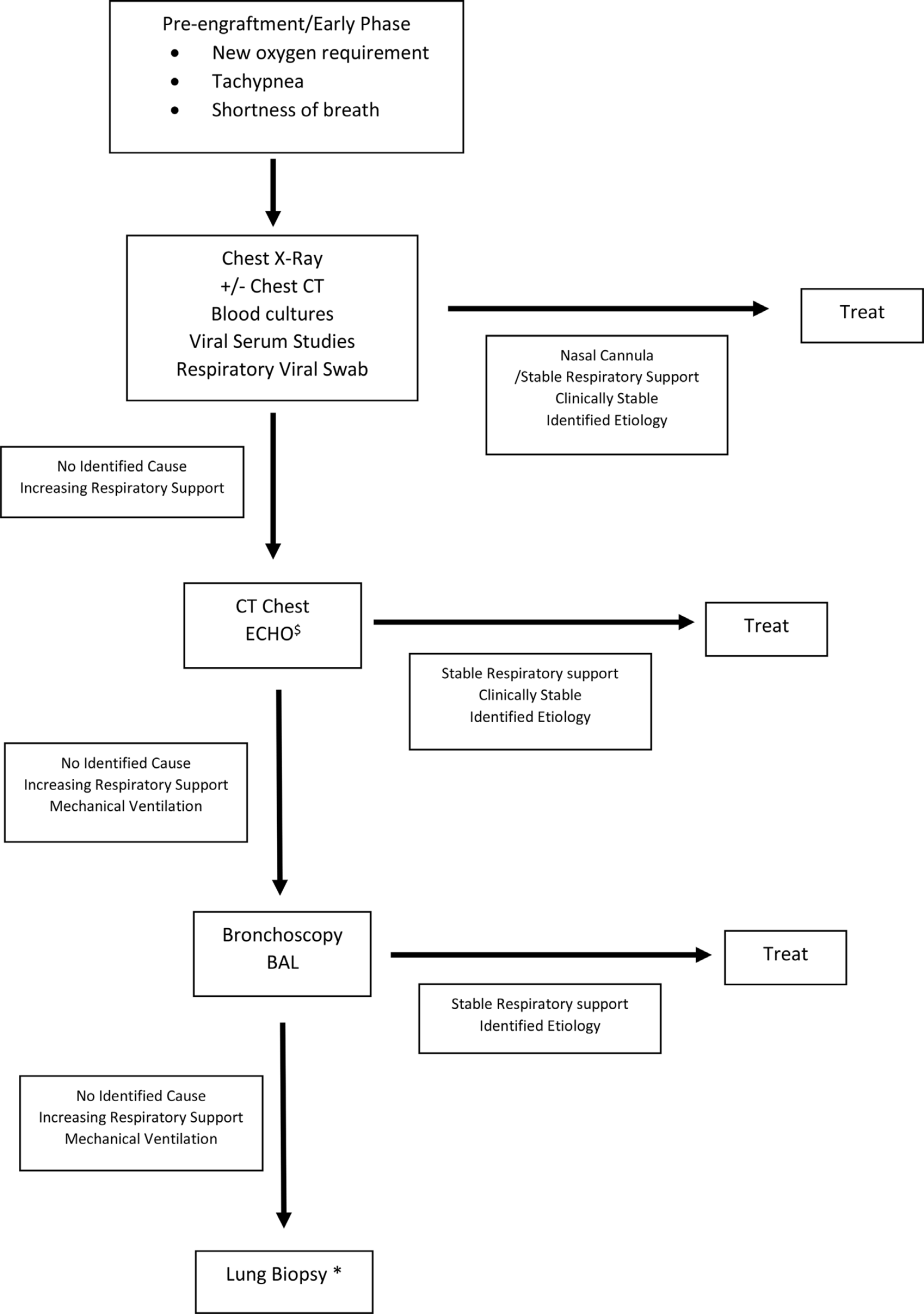

Suggested diagnostic algorithm. Empiric treatment might be indicated depending on clinical status of patient. If patient requires intubation, all studies along the vertical access should be performed. ^$^ECHO to evaluate for pulmonary hypertension, increased pulmonary vascular resistance, and elevated right ventricular pressure *Lung biopsy pending clinical evaluation and assessment of patient’s ability to tolerate procedure.

Fiber-optic bronchoscopies are the next step to fully evaluate the etiology of pneumonitis and are well tolerated. Cytology and infectious studies (bacterial, fungal, and viral) are recommended. Multiple studies evaluating BAL show it can be diagnostic in about 50% of cases and is more likely to identify infectious etiologies compared to serologic testing and lung biopsies (4, 86–88). When BAL is obtained within the first 24 h of symptoms, yield can be as high as 75%; this is likely due to shorter duration of antibiotics (88). Despite this, there have not been consistent data showing that bronchoscopies have improved mortality likely due to skewed data, as only the sickest patients have bronchoscopies performed (4, 17). In the pre-engraftment/early phase, if the patient requires intubation for respiratory needs, at our institution, bronchoscopies are typically performed. The reasoning for this is multifactorial. If infection is suspected, this is confirmatory; if non-infectious complication is suspected or ultimately discovered to be the cause, ruling out concurrent infection is needed, as the treatment for many non-infectious complications involves further immunosuppression with steroids or other immunosuppressants. If developing in the late phase and more chronic in nature, bronchoscopies are carefully planned with input from pulmonology, bone marrow transplant, and anesthesia teams. Of note, a recent study evaluated pediatric patients’ BAL’s microbe-gene profile to see if a subset was more likely to develop pulmonary complications. What was found was that those with significant microbial depletion and concomitant natural killer/T-cell activation have higher incidence of pulmonary complication post-HSCT. This is a new and evolving field that may allow for predicting those with complications and ultimately help develop preventative measures (89).

The next diagnostic step can be a surgical lung biopsy; however, this does come with significant mortality. Biopsy was associated with a 4-fold higher procedure-related mortality of about 8% with common complications being pneumothorax, hemothorax, prolonged mechanical ventilation, and wound dehiscence (23, 86). This procedure has been more successful in identifying non-infectious causes of pneumonitis (86). In one study, a diagnosis was found 62% of the time, with a change in therapy made 57% of the time based on results (90). For those with a specific diagnosis, Day +30 and Day +90 outcomes were improved compared to those without a diagnosis (90). At our institution, lung biopsies are not routinely performed on patients with acute pulmonary deterioration but rather on the patients with chronic or refractory pulmonary disease. The workup mentioned above is almost always completed prior to proceeding with biopsy, and if there are continued clinical concerns or questions, biopsy is performed based on clinical stability of the patient and suspected clinical diagnosis. Unfortunately, there is limited literature to make uniformed recommendations and remains at each clinician’s discretion.

## Long-Term Follow-Up

In less acute phases, pulmonary function testing (PFT) with diffusion capacity [measured by diffusing capacity of the lungs for carbon monoxide (DLCO)] is used as a marker both pre- and post-HSCT to determine the risk of pulmonary complications during HSCT and risk of mortality after HSCT (5, 9, 17, 91, 92). The pre-HSCT PFTs establish a baseline and identify asymptomatic patients who have underlying pulmonary changes. Based on PFT results, further evaluation and adjustments can be made in conditioning regimens (22, 92). Studies have suggested that worsening spirometry measurements and DLCO are usually found immediately after transplant but recover partially then ultimately stabilize (91, 93, 94). When PFTs are abnormal, they typically show a restrictive rather than obstructive pattern (93–95). Studies, though variable, show poor baseline spirometry measurements, more pulmonary complications, and worse post-HSCT spirometry measurements if the patient had total body irradiation, lung metastasis, prior thoracotomy, lung radiation, and previous chemotherapy (9, 91–93, 95).

## Conclusion

Pulmonary complications are common and cause significant morbidity and mortality for HSCT patients. Both allogeneic and autologous HSCT recipients experience these toxicities, though they occur most frequently in allogeneic HSCT patients. The process of the lung injury varies when comparing pre-engraftment, post-engraftment, and late phases and whether the underlying complication is infectious or non-infectious in nature. Recently, the incidence of non-infectious complications has been increasing. Thus, a better understanding of the etiology, evaluation, and treatment of these disorders is needed. Additionally, there are very few studies evaluating autologous HSCT patients and the complications that affect these individuals. Recently, a growing number of patients at our institution experienced chemotherapy-induced pneumonitis, highlighting the need for a better understanding of this complication. Though murine models have helped elucidate a proposed mechanism of injury, this has been much harder to determine in our patients. Additionally, there are no readily available biomarkers or serologic testing that definitively point to chemotherapy-induced pneumonitis. Thus, it remains in large part a diagnosis of exclusion and clinical intuition. Ultimately, improved outcomes for patients with pulmonary complications can be achieved by understanding the underlying etiology and the mechanism of action of the resultant injury and by standardizing the workup and treatment (including duration).

## Author Contributions

TF wrote the first draft and assimilated data. KM contributed to editing the article with each subsequent draft. CT contributed to editing the article. MD helped with the figures and edited subsequent drafts. CD helped with final edits, primary research, and tables. All authors contributed to the article and approved the submitted version.

## Conflict of Interest

The authors declare that the research was conducted in the absence of any commercial or financial relationships that could be construed as a potential conflict of interest.

## Publisher’s Note

All claims expressed in this article are solely those of the authors and do not necessarily represent those of their affiliated organizations, or those of the publisher, the editors and the reviewers. Any product that may be evaluated in this article, or claim that may be made by its manufacturer, is not guaranteed or endorsed by the publisher.

## References

[1] D’Souza A Fretham C Lee SJ Arora M Brunner J Chhabra S. Current Use of and Trends in Hematopoietic Cell Transplantation in the United States. Biol Blood Marrow Transplant (2020) 26(8):e177–82. doi: 10.1016/j.bbmt.2020.04.013 PMC7404814 32438042

[2] Majhail NS Farnia SH Carpenter PA Champlin RE Crawford S Marks DI. Indications for Autologous and Allogeneic Hematopoietic Cell Transplantation: Guidelines From the American Society for Blood and Marrow Transplantation. Biol Blood Marrow Transplant (2015) 21(11):1863–9. doi: 10.1016/j.bbmt.2015.07.032 PMC4830270 26256941

[3] Gooley TA Chien JW Pergam SA Hingorani S Sorror ML Boeckh M. Reduced Mortality After Allogeneic Hematopoietic-Cell Transplantation. N Engl J Med (2010) 363(22):2091–101. doi: 10.1056/NEJMoa1004383 PMC3017343 21105791

[4] Eikenberry M Bartakova H Defor T Haddad IY Ramsay NK Blazar BR. Natural History of Pulmonary Complications in Children After Bone Marrow Transplantation. Biol Blood Marrow Transplant (2005) 11(1):56–64. doi: 10.1016/j.bbmt.2004.09.008 15625545

[5] Afessa B Abdulai RM Kremers WK Hogan WJ Litzow MR Peters SG. Risk Factors and Outcome of Pulmonary Complications After Autologous Hematopoietic Stem Cell Transplant. Chest (2012) 141(2):442–50. doi: 10.1378/chest.10-2889 21778261

[6] Dandoy CE Rotz S Alonso PB Klunk A Desmond C Huber J. A Pragmatic Multi-Institutional Approach to Understanding Transplant-Associated Thrombotic Microangiopathy After Stem Cell Transplant. Blood Adv (2020) 5(1):1–11. doi: 10.1182/bloodadvances.2020003455 PMC7805323 33570619

[7] van Gestel JP Bierings MB Dauger S Dalle JH Pavlíček P Sedláček P. Outcome of Invasive Mechanical Ventilation After Pediatric Allogeneic Hematopoietic SCT: Results From a Prospective, Multicenter Registry. Bone Marrow Transplant (2014) 49(10):1287–92. doi: 10.1038/bmt.2014.147 25068426

[8] Soubani AO Miller KB Hassoun PM. Pulmonary Complications of Bone Marrow Transplantation. Chest (1996) 109(4):1066–77. doi: 10.1378/chest.109.4.1066 8635332

[9] Ghalie R Szidon JP Thompson L Nawas YN Dolce A Kaizer H. Evaluation of Pulmonary Complications After Bone Marrow Transplantation: The Role of Pretransplant Pulmonary Function Tests. Bone Marrow Transplant (1992) 10(4):359–65. 1422492

[10] Afessa B Litzow MR Tefferi A. Bronchiolitis Obliterans and Other Late Onset non-Infectious Pulmonary Complications in Hematopoietic Stem Cell Transplantation. Bone Marrow Transplant (2001) 28(5):425–34. doi: 10.1038/sj.bmt.1703142 11593314

[11] Afessa B Peters SG. Major Complications Following Hematopoietic Stem Cell Transplantation. Semin Respir Crit Care Med (2006) 27(3):297–309. doi: 10.1055/s-2006-945530 16791762

[12] Roychowdhury M Pambuccian SE Aslan DL Jessurun J Rose AG Manivel JC. Pulmonary Complications After Bone Marrow Transplantation: An Autopsy Study From a Large Transplantation Center. Arch Pathol Lab Med (2005) 129(3):366–71. doi: 10.5858/2005-129-366-PCABMT 15737032

[13] Inaba H Yang J Pan J Stokes DC Krasin MJ Srinivasan A. Pulmonary Dysfunction in Survivors of Childhood Hematologic Malignancies After Allogeneic Hematopoietic Stem Cell Transplantation. Cancer (2010) 116(8):2020–30. doi: 10.1002/cncr.24897 PMC2919832 20186702

[14] Chi AK Soubani AO White AC Miller KB. An Update on Pulmonary Complications of Hematopoietic Stem Cell Transplantation. Chest (2013) 144(6):1913–22. doi: 10.1378/chest.12-1708 24297123

[15] Diab M ZazaDitYafawi J Soubani AO. Major Pulmonary Complications After Hematopoietic Stem Cell Transplant. Exp Clin Transplant (2016) 14(3):259–70. doi: 10.6002/ect.2015.0275 27040986

[16] Kumar D Tellier R Draker R Levy G Humar A. Severe Acute Respiratory Syndrome (SARS) in a Liver Transplant Recipient and Guidelines for Donor SARS Screening. Am J Transplant (2003) 3(8):977–81. doi: 10.1034/j.1600-6143.2003.00197.x PMC7175989 12859532

[17] Kaya Z Weiner DJ Yilmaz D Rowan J Goyal RK. Lung Function, Pulmonary Complications, and Mortality After Allogeneic Blood and Marrow Transplantation in Children. Biol Blood Marrow Transplant (2009) 15(7):817–26. doi: 10.1016/j.bbmt.2009.03.019 19539213

[18] Kumar D Humar A Plevneshi A Siegal D Franke N Green K. Invasive Pneumococcal Disease in Adult Hematopoietic Stem Cell Transplant Recipients: A Decade of Prospective Population-Based Surveillance. Bone Marrow Transplant (2008) 41(8):743–7. doi: 10.1038/sj.bmt.1705964 18176614

[19] Folz RJ. Mechanisms of Lung Injury After Bone Marrow Transplantation. Am J Respir Cell Mol Biol (1999) 20(6):1097–9. doi: 10.1165/ajrcmb.20.6.f152 10340926

[20] Horger MS Pfannenberg C Einsele H Beck R Hebart H Lengerke C. Cytomegalovirus Pneumonia After Stem Cell Transplantation: Correlation of CT Findings With Clinical Outcome in 30 Patients. AJR Am J Roentgenol (2006) 187(6):W636–43. doi: 10.2214/AJR.04.1592 17114518

[21] Lim DH Lee J Lee HG Park BB Peck KR Oh WS. Pulmonary Complications After Hematopoietic Stem Cell Transplantation. J Korean Med Sci (2006) 21(3):406–11. doi: 10.3346/jkms.2006.21.3.406 PMC2729942 16778380

[22] Çıkı K Doğru D Kuşkonmaz B Emiralioğlu N Yalçın E Özçelik U. Pulmonary Complications Following Hematopoietic Stem Cell Transplantation in Children. Turk J Pediatr (2019) 61(1):59–60. doi: 10.24953/turkjped.2019.01.010 31559723

[23] Sirithanakul K Salloum A Klein JL Soubani AO. Pulmonary Complications Following Hematopoietic Stem Cell Transplantation: Diagnostic Approaches. Am J Hematol (2005) 80(2):137–46. doi: 10.1002/ajh.20437 16184594

[24] Sadon AA El-Hagrasy R Saraya M. Pulmonary Complications Within the First Year After Bone Marrow Transplantation. Egyptian J Bronchol (2018) 12(2):233–9. doi: 10.4103/ejb.ejb_33_17

[25] Bondeelle L Bergeron A. Managing Pulmonary Complications in Allogeneic Hematopoietic Stem Cell Transplantation. Expert Rev Respir Med (2019) 13(1):105–19. doi: 10.1080/17476348.2019.1557049 30523731

[26] Harris B Geyer AI. Diagnostic Evaluation of Pulmonary Abnormalities in Patients With Hematologic Malignancies and Hematopoietic Cell Transplantation. Clin Chest Med (2017) 38(2):317–31. doi: 10.1016/j.ccm.2016.12.008 PMC7172342 28477642

[27] Daly AS McGeer A Lipton JH. Systemic Nocardiosis Following Allogeneic Bone Marrow Transplantation. Transpl Infect Dis (2003) 5(1):16–20. doi: 10.1034/j.1399-3062.2003.00007.x 12791070

[28] Mansi L Daguindau E Saas P Pouthier F Ferrand C Dormoy A. Diagnosis and Management of Nocardiosis After Bone Marrow Stem Cell Transplantation in Adults: Lack of Lymphocyte Recovery as a Major Contributing Factor. Pathol Biol (Paris) (2014) 62(3):156–61. doi: 10.1016/j.patbio.2014.04.001 24875455

[29] van Burik JA Hackman RC Nadeem SQ Hiemenz JW White MH Flowers ME. Nocardiosis After Bone Marrow Transplantation: A Retrospective Study. Clin Infect Dis (1997) 24(6):1154–60. doi: 10.1086/513654 9195074

[30] Minero MV Marín M Cercenado E Rabadán PM Bouza E Muñoz P. Nocardiosis at the Turn of the Century. Med (Baltimore) (2009) 88(4):250–61. doi: 10.1097/MD.0b013e3181afa1c8 19593231

[31] Russo RL Dulley FL Suganuma L França IL Yasuda MA Costa SF. Tuberculosis in Hematopoietic Stem Cell Transplant Patients: Case Report and Review of the Literature. Int J Infect Dis (2010) 14 Suppl 3:e187–91. doi: 10.1016/j.ijid.2009.08.001 19819176

[32] Akan H Arslan O Akan OA. Tuberculosis in Stem Cell Transplant Patients. J Hosp Infect (2006) 62(4):421–6. doi: 10.1016/j.jhin.2005.09.020 16413085

[33] Eom KS Lee DG Lee HJ Cho SY Choi SM Choi JK. Tuberculosis Before Hematopoietic Stem Cell Transplantation in Patients With Hematologic Diseases: Report of a Single-Center Experience. Transpl Infect Dis (2015) 17(1):73–9. doi: 10.1111/tid.12341 PMC4345421 25620389

[34] Fan WC Liu CJ Hong YC Feng JY Su WJ Chien SH. Long-Term Risk of Tuberculosis in Haematopoietic Stem Cell Transplant Recipients: A 10-Year Nationwide Study. Int J Tuberc Lung Dis (2015) 19(1):58–64. doi: 10.5588/ijtld.14.0301 25519791

[35] Kontoyiannis DP Marr KA Park BJ Alexander BD Anaissie EJ Walsh TJ. Prospective Surveillance for Invasive Fungal Infections in Hematopoietic Stem Cell Transplant Recipients, 2001-2006: Overview of the Transplant-Associated Infection Surveillance Network (TRANSNET) Database. Clin Infect Dis (2010) 50(8):1091–100. doi: 10.1086/651263 20218877

[36] Ullmann AJ Lipton JH Vesole DH Chandrasekar P Langston A Tarantolo SR. Posaconazole or Fluconazole for Prophylaxis in Severe Graft-Versus-Host Disease. N Engl J Med (2007) 356(4):335–47. doi: 10.1056/NEJMoa061098 17251530

[37] Patterson TF Thompson GR 3rd Denning DW Fishman JA Hadley S Herbrecht R. Practice Guidelines for the Diagnosis and Management of Aspergillosis: 2016 Update by the Infectious Diseases Society of America. Clin Infect Dis (2016) 63(4):e1–60. doi: 10.1093/cid/ciw326 27365388 PMC4967602

[38] Bergeron A Porcher R Sulahian A de Bazelaire C Chagnon K Raffoux E. The Strategy for the Diagnosis of Invasive Pulmonary Aspergillosis Should Depend on Both the Underlying Condition and the Leukocyte Count of Patients With Hematologic Malignancies. Blood (2012) 119(8):1831–7. doi: 10.1182/blood-2011-04-351601 22010103

[39] Singh N. Impact of Current Transplantation Practices on the Changing Epidemiology of Infections in Transplant Recipients. Lancet Infect Dis (2003) 3(3):156–61. doi: 10.1016/S1473-3099(03)00546-2 12614732

[40] Girmenia C Finolezzi E Federico V Santopietro M Perrone S. Invasive Candida Infections in Patients With Haematological Malignancies and Hematopoietic Stem Cell Transplant Recipients: Current Epidemiology and Therapeutic Options. Mediterr J Hematol Infect Dis (2011) 3(1):e2011013. doi: 10.4084/mjhid.2011.013 21625317 PMC3103241

[41] Farmakiotis D Kontoyiannis DP. Mucormycoses. Infect Dis Clin North Am (2016) 30(1):143–63. doi: 10.1016/j.idc.2015.10.011 26897065

[42] Williams KM Ahn KW Chen M Aljurf MD Agwu AL Chen AR. The Incidence, Mortality and Timing of Pneumocystis Jiroveci Pneumonia After Hematopoietic Cell Transplantation: A CIBMTR Analysis. Bone Marrow Transplant (2016) 51(4):573–80. doi: 10.1038/bmt.2015.316 PMC4823157 26726945

[43] Meyers JD Flournoy N Thomas ED. Risk Factors for Cytomegalovirus Infection After Human Marrow Transplantation. J Infect Dis (1986) 153(3):478–88. doi: 10.1093/infdis/153.3.478 3005424

[44] Konoplev S Champlin RE Giralt S Ueno NT Khouri I Raad I. Cytomegalovirus Pneumonia in Adult Autologous Blood and Marrow Transplant Recipients. Bone Marrow Transplant (2001) 27(8):877–81. doi: 10.1038/sj.bmt.1702877 11477447

[45] Linder KA Kovacs C Mullane KM Wolfe C Clark NM La Hoz RM. Letermovir Treatment of Cytomegalovirus Infection or Disease in Solid Organ and Hematopoietic Cell Transplant Recipients. Transpl Infect Dis (2021) 23(4):e13687. doi: 10.1111/tid.13687 34251742

[46] Manuel O Avery RK. Update on Cytomegalovirus in Transplant Recipients: New Agents, Prophylaxis, and Cell-Mediated Immunity. Curr Opin Infect Dis (2021) 34(4):307–13. doi: 10.1097/QCO.0000000000000746 34074879

[47] Vakil E Evans SE. Viral Pneumonia in Patients With Hematologic Malignancy or Hematopoietic Stem Cell Transplantation. Clin Chest Med (2017) 38(1):97–111. doi: 10.1016/j.ccm.2016.11.002 28159165 PMC5373482

[48] Seo S Waghmare A Scott EM Xie H Kuypers JM Hackman RC. Human Rhinovirus Detection in the Lower Respiratory Tract of Hematopoietic Cell Transplant Recipients: Association With Mortality. Haematologica (2017) 102(6):1120–30. doi: 10.3324/haematol.2016.153767 PMC5451345 28183847

[49] Markovic SN Adlakha A Smith TF Walker RC. Respiratory Syncytial Virus Pneumonitis-Induced Diffuse Alveolar Damage in an Autologous Bone Marrow Transplant Recipient. Mayo Clin Proc (1998) 73(2):153–6. doi: 10.1016/S0025-6196(11)63648-3 9472999

[50] Hertz MI Englund JA Snover D Bitterman PB McGlave PB. Respiratory Syncytial Virus-Induced Acute Lung Injury in Adult Patients With Bone Marrow Transplants: A Clinical Approach and Review of the Literature. Med (Baltimore) (1989) 68(5):269–81. doi: 10.1097/00005792-198909000-00002 2677595

[51] Peña E Souza CA Escuissato DL Gomes MM Allan D Tay J. Noninfectious Pulmonary Complications After Hematopoietic Stem Cell Transplantation: Practical Approach to Imaging Diagnosis. Radiographics (2014) 34(3):663–83. doi: 10.1148/rg.343135080 24819788

[52] Cengiz Seval G Topçuoğlu P Demirer T. Current Approach to Non-Infectious Pulmonary Complications of Hematopoietic Stem Cell Transplantation. Balkan Med J (2018) 35(2):131–40. doi: 10.4274/balkanmedj.2017.1635 PMC5863250 29553463

[53] Maiolino A Biasoli I Lima J Portugal AC Pulcheri W Nucci M. Engraftment Syndrome Following Autologous Hematopoietic Stem Cell Transplantation: Definition of Diagnostic Criteria. Bone Marrow Transplant (2003) 31(5):393–7. doi: 10.1038/sj.bmt.1703855 12634731

[54] Sheth V Jain R Gore A Ghanekar A Saikia T. Engraftment Syndrome: Clinical Features and Predictive Factors in Autologous Stem Cell Transplant. Indian J Hematol Blood Transfus (2018) 34(3):448–53. doi: 10.1007/s12288-017-0899-4 PMC6081308 30127551

[55] Capizzi SA Kumar S Huneke NE Gertz MA Inwards DJ Litzow MR. Peri-Engraftment Respiratory Distress Syndrome During Autologous Hematopoietic Stem Cell Transplantation. Bone Marrow Transplant (2001) 27(12):1299–303. doi: 10.1038/sj.bmt.1703075 11548849

[56] Karlin L Darmon M Thiéry G Ciroldi M de Miranda S Lefebvre A. Respiratory Status Deterioration During G-CSF-Induced Neutropenia Recovery. Bone Marrow Transplant (2005) 36(3):245–50. doi: 10.1038/sj.bmt.1705037 PMC7092208 15937498

[57] Gerbitz A Nickoloff BJ Olkiewicz K Willmarth NE Hildebrandt G Liu C. A Role for Tumor Necrosis Factor-Alpha-Mediated Endothelial Apoptosis in the Development of Experimental Idiopathic Pneumonia Syndrome. Transplantation (2004) 78(4):494–502. doi: 10.1097/01.TP.0000128839.13674.02 15446306

[58] Panoskaltsis-Mortari A Griese M Madtes DK Belperio JA Haddad IY Folz RJ. An Official American Thoracic Society Research Statement: Noninfectious Lung Injury After Hematopoietic Stem Cell Transplantation: Idiopathic Pneumonia Syndrome. Am J Respir Crit Care Med (2011) 183(9):1262–79. doi: 10.1164/rccm.2007-413ST PMC3266140 21531955

[59] Kantrow SP Hackman RC Boeckh M Myerson D Crawford SW. Idiopathic Pneumonia Syndrome: Changing Spectrum of Lung Injury After Marrow Transplantation. Transplantation (1997) 63(8):1079–86. doi: 10.1097/00007890-199704270-00006 9133468

[60] Yanik GA Ho VT Levine JE White ES Braun T Antin JH. The Impact of Soluble Tumor Necrosis Factor Receptor Etanercept on the Treatment of Idiopathic Pneumonia Syndrome After Allogeneic Hematopoietic Stem Cell Transplantation. Blood (2008) 112(8):3073–81. doi: 10.1182/blood-2008-03-143412 PMC3045267 18664626

[61] Yanik GA Horowitz MM Weisdorf DJ Logan BR Ho VT Soiffer RJ. Randomized, Double-Blind, Placebo-Controlled Trial of Soluble Tumor Necrosis Factor Receptor: Enbrel (Etanercept) for the Treatment of Idiopathic Pneumonia Syndrome After Allogeneic Stem Cell Transplantation: Blood and Marrow Transplant Clinical Trials Network Protocol. Biol Blood Marrow Transplant (2014) 20(6):858–64. doi: 10.1016/j.bbmt.2014.02.026 PMC4128626 24607553

[62] Heggen J West C Olson E Olson T Teague G Fortenberry J. Diffuse Alveolar Hemorrhage in Pediatric Hematopoietic Cell Transplant Patients. Pediatrics (2002) 109(5):965–71. doi: 10.1542/peds.109.5.965 11986464

[63] Fan K McArthur J Morrison RR Ghafoor S. Diffuse Alveolar Hemorrhage After Pediatric Hematopoietic Stem Cell Transplantation. Front Oncol (2020) 10:1757. doi: 10.3389/fonc.2020.01757 33014865 PMC7509147

[64] Afessa B Tefferi A Litzow MR Peters SG. Outcome of Diffuse Alveolar Hemorrhage in Hematopoietic Stem Cell Transplant Recipients. Am J Respir Crit Care Med (2002) 166(10):1364–8. doi: 10.1164/rccm.200208-792OC 12406834

[65] Robbins RA Linder J Stahl MG Thompson AB 3rd Haire W Kessinger A. Diffuse Alveolar Hemorrhage in Autologous Bone Marrow Transplant Recipients. Am J Med (1989) 87(5):511–8. doi: 10.1016/S0002-9343(89)80606-0 2816966

[66] O’Neil ER Schmees LR Resendiz K Justino H Anders MM. Inhaled Tranexamic Acid as a Novel Treatment for Pulmonary Hemorrhage in Critically Ill Pediatric Patients: An Observational Study. Crit Care Explor (2020) 2(1):e0075. doi: 10.1097/CCE.0000000000000075 32166295 PMC7063899

[67] Bafaqih H Chehab M Almohaimeed S Thabet F Alhejaily A AlShahrani M. Pilot Trial of a Novel Two-Step Therapy Protocol Using Nebulized Tranexamic Acid and Recombinant Factor VIIa in Children With Intractable Diffuse Alveolar Hemorrhage. Ann Saudi Med (2015) 35(3):231–9. doi: 10.5144/0256-4947.2015.231 PMC6074453 26409798

[68] Lane AA Armand P Feng Y Neuberg DS Abramson JS Brown JR. Risk Factors for Development of Pneumonitis After High-Dose Chemotherapy With Cyclophosphamide, BCNU and Etoposide Followed by Autologous Stem Cell Transplant. Leuk Lymphoma (2012) 53(6):1130–6. doi: 10.3109/10428194.2011.645208 PMC3376378 22132836

[69] Bhalla KS Wilczynski SW Abushamaa AM Petros WP McDonald CS Loftis JS. Pulmonary Toxicity of Induction Chemotherapy Prior to Standard or High-Dose Chemotherapy With Autologous Hematopoietic Support. Am J Respir Crit Care Med (2000) 161(1):17–25. doi: 10.1164/ajrccm.161.1.9903059 10619792

[70] Wells AU. Cryptogenic Organizing Pneumonia. Semin Respir Crit Care Med (2001) 22(4):449–60. doi: 10.1055/s-2001-17387 16088692

[71] Freudenberger TD Madtes DK Curtis JR Cummings P Storer BE Hackman RC. Association Between Acute and Chronic Graft-Versus-Host Disease and Bronchiolitis Obliterans Organizing Pneumonia in Recipients of Hematopoietic Stem Cell Transplants. Blood (2003) 102(10):3822–8. doi: 10.1182/blood-2002-06-1813 12869516

[72] Dandoy CE Hirsch R Chima R Davies SM Jodele S. Pulmonary Hypertension After Hematopoietic Stem Cell Transplantation. Biol Blood Marrow Transplant (2013) 19(11):1546–56. doi: 10.1016/j.bbmt.2013.07.017 23891748

[73] Jodele S Hirsch R Laskin B Davies S Witte D Chima R. Pulmonary Arterial Hypertension in Pediatric Patients With Hematopoietic Stem Cell Transplant-Associated Thrombotic Microangiopathy. Biol Blood Marrow Transplant (2013) 19(2):202–7. doi: 10.1016/j.bbmt.2012.08.022 22960385

[74] Levy M Moshous D Szezepanski I Galmiche L Castelle M Lesage F. Pulmonary Hypertension After Bone Marrow Transplantation in Children. Eur Respir J (2019) 54(5):1900612. doi: 10.1183/13993003.00612-2019 31649064

[75] Rabinovitch M. Molecular Pathogenesis of Pulmonary Arterial Hypertension. J Clin Invest (2012) 122(12):4306–13. doi: 10.1172/JCI60658 PMC3533531 23202738

[76] Montani D O’Callaghan DS Savale L Jaïs X Yaïci A Maitre S. Pulmonary Veno-Occlusive Disease: Recent Progress and Current Challenges. Respir Med (2010) 104 Suppl 1:S23–32. doi: 10.1016/j.rmed.2010.03.014 20456932

[77] Seguchi M Hirabayashi N Fujii Y Azuno Y Fujita N Takeda K. Pulmonary Hypertension Associated With Pulmonary Occlusive Vasculopathy After Allogeneic Bone Marrow Transplantation. Transplantation (2000) 69(1):177–9. doi: 10.1097/00007890-200001150-00030 10653399

[78] Barker AF Bergeron A Rom WN Hertz MI. Obliterative Bronchiolitis. N Engl J Med (2014) 370(19):1820–8. doi: 10.1056/NEJMra1204664 24806161

[79] Jagasia MH Greinix HT Arora M Williams KM Wolff D Cowen EW. National Institutes of Health Consensus Development Project on Criteria for Clinical Trials in Chronic Graft-Versus-Host Disease: I. The 2014 Diagnosis and Staging Working Group Report. Biol Blood Marrow Transplant (2015) 21(3):389–401.e1. doi: 10.1016/j.bbmt.2014.12.001 25529383 PMC4329079

[80] Escamilla Gómez V García-Gutiérrez V López Corral L García Cadenas I Pérez Martínez A Márquez Malaver FJ. Ruxolitinib in Refractory Acute and Chronic Graft-Versus-Host Disease: A Multicenter Survey Study. Bone Marrow Transplant (2020) 55(3):641–8. doi: 10.1038/s41409-019-0731-x PMC7051903 31700138

[81] Zeiser R Polverelli N Ram R Hashmi SK Chakraverty R Middeke JM. Ruxolitinib for Glucocorticoid-Refractory Chronic Graft-Versus-Host Disease. N Engl J Med (2021) 385(3):228–38. doi: 10.1056/NEJMoa2033122 34260836

[82] Zhao Y OuYang G Shi J Luo Y Tan Y Yu J. Salvage Therapy With Low-Dose Ruxolitinib Leads to a Significant Improvement in Bronchiolitis Obliterans Syndrome in Patients With Cgvhd After Allogeneic Hematopoietic Stem Cell Transplantation. Front Pharmacol (2021) 12:668825. doi: 10.3389/fphar.2021.668825 34262450 PMC8273229

[83] Wang YM Teusink-Cross A Elborai Y Krupski MC Nelson AS Grimley MS. Ruxolitinib for the Treatment of Chronic GVHD and Overlap Syndrome in Children and Young Adults. Transplantation (2021). doi: 10.1097/TP.0000000000003768 33795598

[84] Cutler CS Lee SJ Arai S Rotta M Zoghi B Lazaryan A. Belumosudil for Chronic Graft-Versus-Host Disease (Cgvhd) After 2 or More Prior Lines of Therapy: The Rockstar Study. Blood (2021) blood.2021012021. doi: 10.1182/blood.2021012021 34265047 PMC8641099

[85] Jaglowski SM Blazar BR. How Ibrutinib, a B-Cell Malignancy Drug, Became an FDA-Approved Second-Line Therapy for Steroid-Resistant Chronic GVHD. Blood Adv (2018) 2(15):2012–9. doi: 10.1182/bloodadvances.2018013060 PMC6093735 30108109

[86] Chellapandian D Lehrnbecher T Phillips B Fisher BT Zaoutis TE Steinbach WJ. Bronchoalveolar Lavage and Lung Biopsy in Patients With Cancer and Hematopoietic Stem-Cell Transplantation Recipients: A Systematic Review and Meta-Analysis. J Clin Oncol (2015) 33(5):501–9. doi: 10.1200/JCO.2014.58.0480 25559816

[87] Dunagan DP Baker AM Hurd DD Haponik EF. Bronchoscopic Evaluation of Pulmonary Infiltrates Following Bone Marrow Transplantation. Chest (1997) 111(1):135–41. doi: 10.1378/chest.111.1.135 8996007

[88] Shannon VR Andersson BS Lei X Champlin RE Kontoyiannis DP. Utility of Early Versus Late Fiberoptic Bronchoscopy in the Evaluation of New Pulmonary Infiltrates Following Hematopoietic Stem Cell Transplantation. Bone Marrow Transplant (2010) 45(4):647–55. doi: 10.1038/bmt.2009.203 19684637

[89] Zinter MS Lindemans CA Versluys BA Mayday MY Sunshine S Reyes G. The Pulmonary Metatranscriptome Prior to Pediatric HCT Identifies Post-HCT Lung Injury. Blood (2021) 137(12):1679–89. doi: 10.1182/blood.2020009246 PMC7995292 33512420

[90] White DA Wong PW Downey R. The Utility of Open Lung Biopsy in Patients With Hematologic Malignancies. Am J Respir Crit Care Med (2000) 161(3 Pt 1):723–9. doi: 10.1164/ajrccm.161.3.9904016 10712314

[91] Carlson K Bäcklund L Smedmyr B Oberg G Simonsson B. Pulmonary Function and Complications Subsequent to Autologous Bone Marrow Transplantation. Bone Marrow Transplant (1994) 14(5):805–11. 7889014

[92] Chien JW Madtes DK Clark JG. Pulmonary Function Testing Prior to Hematopoietic Stem Cell Transplantation. Bone Marrow Transplant (2005) 35(5):429–35. doi: 10.1038/sj.bmt.1704783 15654355

[93] Frisk P Arvidson J Bratteby L-E Hedenström H Lönnerholm G. Pulmonary Function After Autologous Bone Marrow Transplantation in Children: A Long-Term Prospective Study. Bone Marrow Transplant (2004) 33(6):645–50. doi: 10.1038/sj.bmt.1704393 14688819

[94] Wieringa J van Kralingen KW Sont JK Bresters D. Pulmonary Function Impairment in Children Following Hematopoietic Stem Cell Transplantation. Pediatr Blood Cancer (2005) 45(3):318–23. doi: 10.1002/pbc.20304 15747333

[95] Nenadov Beck M Meresse V Hartmann O Gaultier C. Long-Term Pulmonary Sequelae After Autologous Bone Marrow Transplantation in Children Without Total Body Irradiation. Bone Marrow Transplant (1995) 16(6):771–5. 8750268

